# Constructing digital assets through blockchain technologies? Unpacking the techno-economic configuration of non-fungible tokens

**DOI:** 10.1177/03063127241286447

**Published:** 2024-11-14

**Authors:** Alia Miroshnichenko, Kean Birch

**Affiliations:** 1 Independent Scholar, Toronto, ON, Canada; 2 Institute for Technoscience and Society, York University, Toronto, ON, Canada

**Keywords:** non-fungible tokens, NFT, blockchain, digital asset, assetization, tokenization

## Abstract

Non-fungible tokens (NFTs) are novel techno-economic configurations underpinned by cryptocurrency ledgers that transform digital files like graphic art, music, videos, etc. into digital assets. NFTs are often framed as a way for artists and other creators to profit from their activities, transforming ‘experiences’ into something for sale. As such, NFTs raise some questions pertinent to science and technology studies and political economy. We focus on analysing how NFTs are constructed as digital assets by unpacking the practices, devices, relations, and rights implicated in their construction. We use the concept of ‘assetization’ to examine the contingencies, problematics, and implications of NFTs and the claims, practices, and entitlements that configure them as a new type of asset. We undertake this analysis through a research-creation process by summarizing and discussing the process of creating and submitting an NFT to a specialized marketplace.

Non-fungible tokens (NFTs) are presented as novel and unique (i.e., non-fungible) digital assets underpinned by cryptocurrency ledgers that transform digital files like graphic art, music, videos, etc. into techno-economic objects. NFTs are a new kind of asset, in that they are used to transform our ‘experiences’ into something ownable and capitalizable via a range of techno-economic practices, relations, and claims (Russell, 2022). NFTs have been promoted by celebrity figures in the music industry, who believe that NFTs are the future of artist-fan relationships. Snoop Dogg, Grimes, Cardi B, Ozzy Osborne, and many others in the music industry create, sell, and benefit financially from their associated NFT collections (see Abbate et al., 2022). In 2021, multimedia giant Warner Music invested US$11.2 million in Dapper Labs—creators of the NFT collection *CryptoKitties*—to develop new ways for monetizing artistic assets (Castillo, 2021). In the same year, large US labels and talent agencies such as United Talent Agency (UTA), which feature artists such as Florence and The Machine and Pink Floyd, started signing NFT start-ups (Chan, 2022). Media stories about sensational sales of NFT art, like Beeple’s sale of multiple artworks as NFTs for US$69 million, have led to significant interest in their potential for capturing virtual or intangible activities and experiences (O’Dwyer, 2023).

The cryptocurrency market crash in the summer of 2022 led to a collapse in the NFT market. However, NFTs continue to underpin a major transformation of the ‘experience economy’ evident in the expansion of Web3 and the ‘metaverse’.

Digitalized things (e.g., music transformed into a digital file) have been sold on various marketplaces and platforms since the marketization of the internet in the early 2000s. Bodó et al. (2022) argue that both early internet culture and present-day Web3 are characterized by tensions arising from the distribution of digitalized things. Since a digitalized thing is easily copyable and distributable by design, the early internet was often dominated by piracy and copyright infringement issues. Like the ecosystem of the early internet, Bodó et al. (2022) argue that Web3 can also be characterized by concerns with property rights, although this time with the ownership of specifically ‘digital’ things (e.g., virtual plots of land), as opposed to digitalized derivatives (e.g., digitalized music), and especially with greater concern for the emergence of ‘digital’ asset classes (Downling, 2021).

With Web3, new forms of technologically enabled property rights and ownership are emerging through blockchain technologies, especially after establishment of standards such as ERC-721 for non-fungible tokens by ethereum.org (Lotti, 2019). This standard enables people to publicly trace and claim ownership of a digital thing as an asset and has become a defining factor in the popularity of NFTs. Blockchain technologies themselves rose in popularity by allowing so-called ‘trustless’ systems for the decentralized trading of digital tokens (Bodó, 2021; Bratspies, 2018), which are often perceived as financial assets that sit outside government regulations or government-appointed intermediaries.

How are NFTs constructed and configured as assets? How do NFTs differ from other assets in their construction and configuration? And what are the implications of these potential differences? To undertake this analysis, we draw on the concept of ‘assetization’ developed in recent science and technology studies (STS) and cognate literature (Birch, 2017; Birch & Muniesa, 2020; Tellman et al., 2024) to understand the contingencies of contemporary technoscientific capitalism and the construction and governance of NFTs as digital assets. We address the above questions by analyzing how NFTs are constructed as digital assets through techno-economic claims, practices, processes, and governance mechanisms that configure them as particular economic objects, as well as how this process differs (or not) from the construction of other assets. Our analysis is partially based on research-creation, where we summarize and analyze the process of creating and submitting an NFT to a specialized marketplace, as well as a range of secondary sources dealing with NFTs.

## Is there something different about digital assetization?

An increasing number of STS scholars are examining various aspects of assetization, or the transformation of things into assets, such as the transformation of scientific knowledge into intellectual property (Birch, 2017; Birch & Tyfield, 2013; Lezaun & Montgomery, 2015; Martin, 2015), the transformation of work into human capital (Nappert & Plante, 2023), and the transformation of various natural resources into assets (Birch & Muniesa, 2020; Muniesa et al., 2017). More recently, assetization has been used to analyse the emergence and implications of various data assets, including health data (Prainsack, 2020; Vezyridis & Timmons, 2017), agricultural data (Hackfort et al., 2024), digital data (MacKenzie, 2018), and personal data (Beauvisage & Mellet, 2020; Birch, 2023a; Birch et al., 2021).

Building on this work, we argue that digital assets, exemplified by NFTs, represent a distinct asset form. To start, digital assets such as NFTs are implicated in a range of technologically enabled attempts to transform our social lives and individual experiences into capitalizable property. Russell (2022) and others conceptualize this as an ‘experience economy’ in which the production of collective and shared experiences—including digital events (e.g., online concerts) and collectively meaningful shared activities (e.g., trading of digital items)—can be turned into an asset through NFTs (see Boltanski & Esquerre, 2016). As for other assets, stories, imaginaries, and myths play a critical role in generating demand for digital items or activities (Beckert, 2016; Birch, 2023b). Perhaps even more than many other kinds of assets, digital assets are not easily separable from the stories, imaginaries, and myths that support them. Nor are they separable from the digitally mediated social relations or communities constituted by these emotional and experiential connections (e.g., shared interests) and the collective meaning and aesthetics that underpin these ‘experiences’.

Blockchain technologies that configure digital assets like NFTs are premised on the idea of ‘trustless technologies’ (Bratspies, 2018); they do not require social or communal legitimation and enforcement to define and constitute property or other rights. As Bodó (2021) notes, however, decentralized systems like blockchain still rely on social trust, even if third parties and intermediaries involved in the asset governance are not immediately apparent. The creation, distribution, and valuation of NFTs (and other digital assets) are orchestrated by a range of social actors whose actions are neither necessarily transparent nor regulated.

Blockchain technologies are characterized as ‘confidence machines’, technical devices that can replace social or communal relations and trust (De Filippi et al., 2020). As scholars (Callon, 2021; Çalışkan, 2023; Nessel, 2022) have pointed out, trust underpins many of our economic activities and is constituted by collective social relations. ‘Trustless’ technologies, however, are meant to replace these relations with an array of new digital devices and algorithmically generated metrics (e.g., recommendations, rankings; Fourcade & Healy, 2024), all underpinned by technically defined claims and entitlements (Callon, 2021; Pistor, 2019).

Digital assets are different than other assets with regards to the need for property rights, specifically in terms of the *form* that those property rights take and their implications. Some argue that blockchain technologies have ‘instituted’ digital property rights ‘without any form of state intervention or legal enforcement by a third party’ (Rotta & Parana, 2022, p. 1048). And that blockchain technologies (seemingly):

eliminate the need for a third-party authority to delineate and enforce property rights, as these are replaced by network consensus rules that prescribe rights and obligations of network participants, and guarantee their implementations. (Pesch & Ishmaev, 2019, p. 269)

However, what makes digital assets distinct is the form that property and ownership rights take, in that they are characterized by contractual forms of control (see Birch & Muniesa, 2020), which has two important implications.

First, digital assetization creates new possibilities for contractually splitting entitlements through fractional control via ‘tokenization’ (Belk et al., 2022). For O’Dwyer (2020, p. 15), tokenization is a mechanism to make rights ‘transferable but not infinitely replicable’, creating scarcity where it need not exist (e.g. by limiting the number of times that derivatives of a copyrighted asset can be sold). She goes on to argue that tokenization can split ‘the right to profit from the title to a good’ from the exclusion right defined in property law. While fractional control is possible for most other assets, tokenization does not require specialized institutions (e.g. brokerages), experts (e.g., lawyers), or knowledge (e.g., of securities regulators) (see Loepfe & Naegeli, 2024). Second, there is a liminal stage in digital assetization before contractual control is digitally instituted, during which period a digital thing is *becoming* an asset but has not yet been recorded on a blockchain. As such, a digital thing can exist before it is minted, recorded on a blockchain, and (potentially) sold. Here, a digital thing exists in a *pre-asset* state, defined by an intention to create future contractual claims but where it is the act of selling that actually turns it into an asset.

As their distinctive features suggest, digital assets necessarily entail distinct forms of governance. Assetization itself can be thought of as both a mode of governance and a transformative process (e.g. Birch, 2024), as it changes how a resource is understood and organized. This often involves a shift in how it is valued (e.g., capitalized in the present) and knock-on effects on how it is managed over time (e.g. locking-in future policies to achieve future expectations) (Chiapello, 2019; Muniesa et al., 2017; Tellmann, 2022). Moreover, the organization of an asset has a direct impact on its valuation; assetization is an active, ongoing, and reflexive process that reconfigures how something is governed (Birch, 2017). For example, their governance structures often define the value of digital assets through rarity. This is a deliberate technical achievement, as fractional ownership claims—the number of tokens used to define ownership of a digital thing—end up constituting its potential valuation (i.e., the fewer tokens, the higher their valuation). As such, blockchain technologies are used in lieu of intellectual and other property rights, since they come to define how a digital thing is governed via control rights characterized as a restriction of use enforced through ‘smart’ contracts.

## Constructing digital assets through blockchain technologies

Blockchain technologies are usually defined as distributed and decentralized ledgers which digitally record information about past operations and transactions, attaching that information cryptographically to each successive ‘block’ (i.e. record) in the chain (Çalışkan, 2020; Law Commission, 2024; Nabben, 2023; Rella, 2021). Each block is a public and permanent information record, which is extremely difficult to change – making the chain of records secure and trustable. Records can be altered, but this leads to splits or forks in the chain, thereby creating new blockchains. The parties involved in operations or transactions are usually identified via a digital identifier rather than their name, providing anonymity. Blockchain technologies run on a network of computers that both validate transactions and coordinate those transactions across the ledger for participants. This computer network is often run by a community of peers who have agreed to adhere to a specific protocol underpinning the process of adding and validating new blocks.

Blockchain technologies have gone through a series of phases. Çalışkan (2020, 2023) argues that blockchains have evolved since they first emerged with the publication of the 2008 Bitcoin white paper by the pseudonymous Satoshi Nakamoto. First-generation blockchains are associated with cryptocurrencies like Bitcoin, which are designed to transfer monetary tokens (or ‘data money’ – Çalışkan, 2023) without third-party intermediaries, especially intermediaries associated with centralized financial payment or clearing systems (e.g. traditional banks). Second-generation blockchains are often associated with Ethereum, established in 2013, and concern the establishment of contractual conditions through ‘a short computer program’ (Çalışkan, 2020, p.8). This change enabled a reimagining of blockchain functionality, highlighting the role it could play in contractual arrangements; this relates to the creation of ‘smart contracts’ that set conditions for specific effects (Pesch & Ishmaev, 2019). It also meant that ‘off-chain events’ (from the real-world) could be included in blockchain arrangements. Finally, although of less relevance to our discussion here, third-generation blockchains opened up the technology to ‘more complex transactional relations and infrastructure-making’ between chains (Çalışkan, 2020, p.9). Nabben (2023) provides a longer history of blockchain technology, going back to the emergence of public key cryptography in the 1970s.

Cryptocurrencies are digital currencies designed to prevent double spending by using blockchain technology for the communal recording and validation of transactions. As Çalışkan (2020) notes, cryptocurrencies are not necessarily public, although they are necessarily embedded within group interactions and dynamics. Cryptocurrencies function through the cryptographic hash that is recorded on each block in the chain, and which provides public information about past transactions (e.g. timestamps). Unlike non-digital currencies, cryptocurrencies are often created through digital ‘mining’ operations which are defined by a communal protocol: Mining requires computing capacity to solve cryptographic puzzles with solutions leading to the reward of a crypto-coin (O’Dwyer, 2023). Over time, the computing resources needed to mine crypto-coins increase as it becomes deliberately harder and harder to solve the puzzles. Bitcoin was designed to so that ‘the amount of Bitcoin [rewarded] per each block mined would be halved after every 210,000 blocks’ as well as limiting the total number of Bitcoins to only 21 million minable coins (Çalışkan, 2020: 7).

Cryptocurrencies are generally fungible, in that each crypto-coin’s value is interchangeable with the equivalent coin, and its value is not determined by who mines or trades it. Cryptocurrencies, however, are not the same as fiat currencies; as Çalışkan (2023) puts it, they are ‘computationally made’. While some people have sought to treat cryptocurrencies as commodities or securities, they do not neatly fit into either classification and are probably better understood as an alternative asset class (Law Commission, 2024). Generally, cryptocurrencies are considered to be volatile and speculative investments because their value as assets or even transactional processes are not backed up or insured by centralized financial institutions (e.g. clearing banks). Their speculative nature is evident in the fact that their value can change significantly in a short period of time; for example, in the summer of 2022 the ‘cryptocurrency crash’ wiped out over 60% of the market value of various cryptocurrencies, causing investors to lose over US$2 trillion (Ponciano, 2022).

Non-fungible tokens (NFT) followed from second-generation blockchains, starting with the launch of Ethereum in 2013 by Vitalik Buterin (Brody & Couture, 2021; Faustino et al., 2022). Ethereum enabled the programming of self-executing contracts on a blockchain which meant the technology could go beyond the creation of non-standard currencies to the configuration of techno-economic relations and arrangements. Second-generation blockchains have facilitated digital assetization through the contractual configuration of ownership based on standards such as ERC-721, which spells out that:

A Non-Fungible Token (NFT) is used to identify something or someone in a unique way. This type of Token is perfect to be used on platforms that offer collectible items, access keys, lotteray tickets, numbered seats for concerts and sports matches, etc. This special type of Token has amazing possibilities so it deserves a proper Standard, the ERC-721 came to solve that! (ethereum.org, 2023)

NFTs are ‘tokens’ that have unique digital identifiers, making them non-fungible. Tokens can act as an ownership claim on different things, including digital files such as images, music, videos, etc. As digital files are copyable, NFTs can be used to place ownership claims on digital derivatives by pairing a fungible digital object (e.g. image) with a non-fungible blockchain code.

NFTs do not necessarily match up with more conventional property rights, like real estate or intellectual property, although they can be aligned. Rather, an NFT owner owns a digital token but does not necessarily own the underlying property (e.g. copyright). For example, they might own a digital copy of an image, equivalent to owning a physical print of an artwork but not the copyright of the image itself; however, the NFT may give their owner certain use or benefit rights on top of this. As such, an NFT owner might own digital tokens that give them a contractual claim on revenues generated by a physical object or intellectual property without any claim to the other rights that usually go along with property (Rella, 2021). This is because NFTs comprise executable programmes on Ethereum, which enable the creation of contractual arrangements that can extend claims and entitlements in new ways. As with cryptocurrencies. NFTs are publicly validated by a decentralized blockchain, but this time as unique things, which enables the buying, selling, and trading of ownership claims as digital assets.

NFTs gained significant attention for a short time in the late 2010s and early 2020s. A forerunner of NFTs was created in May 2014 at a New York conference and sold during a live presentation, although it was called a ‘monetized graphic’ rather than NFT (Exmundo, 2023). According to etheria.world, the first NFT project was Etheria which was launched in 2015 at Ethereum’s first developer conference, called DEVCON 1 (Etheria World, 2015). Etheria was composed of 457 purchasable and tradable hexagonal digital tiles; these remained unsold until 2021 when growing interest in NFTs led to them selling out for US$1.4 million (NFT Price Floor, 2022). NFT trading took off with the creation of digital collectibles, like the CryptoKitties game created by Canadian firm Dapper Labs in 2017. Ostensibly a blockchain game, CryptoKitties enables players to buy, breed, and trade digital cartoon cats, each with their own unique characteristics. In 2018, CryptoKitties achieved some fame when one rare version was sold at auction for US$140,000 (O’Dwyer, 2023). Today, there are over 1.8 million digital cats and total turnover reached over US$27 million (Rella, 2021).

The popularity of NFTs really took off in 2021. Although it is difficult to specify when the first public NFT marketplaces appeared, the first marketplace to surpass US$1 billion dollars in monthly transactions was OpenSea in mid-2021 (Manoylov, 2021; The Block, 2021). Operating since 2017, OpenSea’s transaction volume increased significantly during the COVID pandemic, peaking in the third quarter of 2021 when monthly trading went from US$284.26 million in July to US$3.16 billion in August (The Block, 2021). Nadini et al.’s (2021) study of trends in NFT markets shows that between 2018 and 2021, the most traded NFTs were ‘games’ (38%) and ‘collectibles’ (40%) – and not ‘art’. As their study further shows, most NFT trading, collecting, and ownership is highly concentrated with the top 10% of NFT traders performing 85% of all transactions. By 2022, the top selling NFT collections (e.g. CryptoPunks, Bored Ape Yacht Club, Mutant Ape Yacht Club, etc.) are mostly owned by or affiliated with the firm Yuga Labs, which created the Bored Ape Yacht Club collection and bought out other collections from other firms (Beyer, 2022).

## Unpacking the techno-economic configuration of NFTs

We now turn to the techno-economic configuration of NFTs as digital assets, including an examination of the tensions and contradictions with other, non-digital asset forms. We focus on four specific issues: the value and valuation of NFTs, the role of trust and framing of blockchain technologies as trustless technologies, the ownership and property rights of digital assets, and how digital assets are governed.

### NFT value and valuation

In ‘How to sell NFTs without really trying’, Frye (2022) describes the process of minting, submitting, and selling NFTs. His first NFT was a stock photo of a Brooklyn Bridge with the subtitle ‘I have a bridge to sell you’, which Frye (2022, p.131) successfully sold: ‘When someone bought my NFT for $100, I was absolutely delighted. I felt like a true 21st-century grifter’. Frye relates his NFT experiments as conceptual art; notably, he did not have to own a copyright or distribution rights to the stock photo to make a sale. In this NFT transaction, no original value beyond ‘experience’ (of buying and owning the NFT) was produced or even implied.

**Figure 1.**
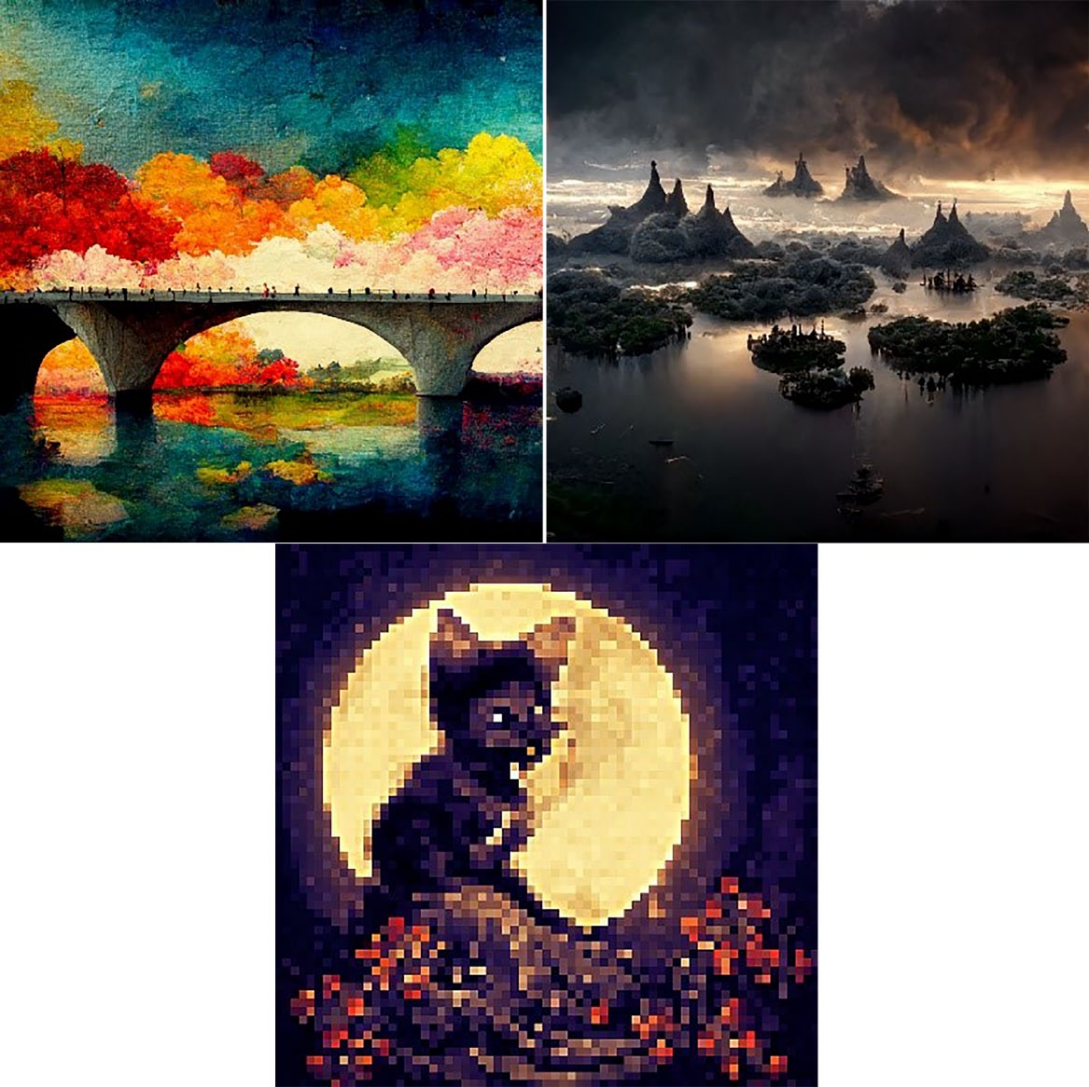
Artworks as digital assets.

In order to familiarize ourselves with this process of NFT creation and valuation, and to test out Frye’s approach of ‘selling NFTs without really trying’, we decided to create several artworks that we could turn into NFTs (see Figure 1). (Although we use the pronoun ‘we’ in this discussion to outline this research-creation process, the actual process of minting the NFT was undertaken by the first author.) For this NFT experiment, we created three small square artworks (1000 x 1000 px each). Our first artwork was a colorful image of a bridge as an homage to Frye’s Brooklyn Bridge. For our second piece, we wanted to create something that could be of a potential value to an NFT enthusiast. We settled on the piece depicting several disconnected (decentralized) islands in the sunrise and named it *Decentraland. Decentraland* is widely known in the NFT ecosystem as one of the most popular virtual real-estate plot collections. We prefaced the artwork with the subtitle ‘The land of opportunities’. Our third and final work was a pixelated image of a cat (because internet users love cats) which we prefaced with ‘selling a soul of this cat’ for a more dramatic effect. Once we had images, our artworks were ready to be turned into digital assets, or ‘assetized’.

For our NFT experiment, we chose to mint our NFTs using Mintable Marketplace, the same marketplace that Frye used. Unlike more popular NFT marketplaces, Mintable does not charge seller transaction fees (also known as ‘gas money’, payable in cryptocurrency), so we could experiment with different concepts without owning any cryptocurrency ourselves. To submit (‘mint’) an NFT, the NFT seller first needs a MetaMask account. MetaMask is a virtual wallet for cryptocurrencies that is tied to the Ethereum blockchain. Most NFTs are minted on the Ethereum blockchain, considered more of a ‘contract-exchange-blockchain’ (Çalışkan, 2023). Creating a MetaMask account did not require us to have any specialized knowledge or understanding of how the wallet is set up; following simple step-by-step user interface prompts was enough.

MetaMask is one of the hundreds of decentralized third-party intermediaries that invites users to trust them with their blockchain transactions. At least in the public-facing space occupied by NFTs, blockchain technologies are dominated by these intermediaries which enable non-technical consumers to engage with digital assets. For example, in our creation of an NFT, we were dependent upon a relatively small number of third parties, including Mintable Marketplace and MetaMask. Consequently, while blockchain technologies might aim to decentralize and distribute control and oversight of digital assets, this process of digital assetization depends upon technical skill and expertise that are not commonly held (see Birch and Muniesa, 2020). Consequently, third parties have inevitably emerged and come to dominate the NFT economy.

The value and valuation of NFTs is rife with a series of tensions and contradictions. One such tension is that by default NFT markets favour early adopters and rely upon a consistent flow of new adopters to ensure liquidity in the marketplace. Early adopters benefit from buying low and selling high when their NFT investment takes off, often as a result of an NFT’s embedding in the stories, imaginaries, and myths promoted and promulgated around the marketplace (Beckert, 2016). As with other collectibles discussed by Boltanski and Esquerre (2016), the valuation of NFTs is a collective achievement in which early adopters benefit by actively enrolling later adopters through stories, etc. So, while there is an emphasis on blockchain technologies being ‘trustless’ (discussed below), largely by reducing social and collective action, NFT markets are dependent upon the construction of a like-minded community to generate trades needed for valuation. While this might seem similar to a Ponzi scheme, it also reflects the ‘normal’ emergence of a market as outlined by Callon (2021): For example, it entails the establishment of specific market sites, relations, encounters, mechanisms of competition, and conventions. As such, NFTs are not automatically assets or even valuable, but must be made so, and that achievement is social *and* technical, involving a range of social actors like digital wallet companies, crypto influencers, exchange platforms, online communities, etc. (Birch & Muniesa, 2020; also Çalışkan 2023).

Following the oversaturation of the NFT market in 2022 caused by an influx of artists and others attempting to monetize digital things, the market shifted towards assetizing ‘experience’. NFT developers started focusing on the social relations, community membership, and experiences that NFTs enabled. This included enrolling celebrities and social media influencers to help boost a collection’s prestige and promotional reach. Non-celebrity enthusiasts also assumed the role of unofficial ambassadors in exchange for rewards. NFT collections that can mobilize these diverse communities can quickly sell out upon launch, as illustrated by Mazur (2021), who shows how NFT startups with impressive first day sales also generate stable consecutive profits. But what this financial marketplace data usually does not account for is the marketing ‘shadowork’ that goes into these launches, including private presales and ‘whitelists’ (see Callon, 2021 on marketing).

**Figure 2.**
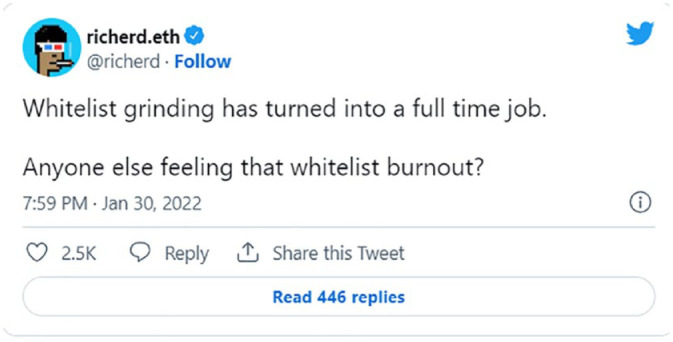
Tweet about grinding.

One example of this shadowork is the tactic of selling out collections before the public release, defined as a ‘two-tiered sale system’ (Genc, 2022). People are will be offered NFTs before public release and can then make a profit by selling them onwards after they are listed on secondary marketplaces. Initial pre-sales are often orchestrated via decentralized Discord community servers, where the collection creators generate social engagement with contests, raffles, and reward for the most active and enthusiastic members. Being ‘whitelisted’, as these perks are called, guarantees potential buyers a presale spot, but access to the whitelist comes at the price of generating hype for the project. According to Genc (2022), ‘whitelist requirements have recently become so exacting and time-consuming that many NFT traders refer to the process as *grinding*’ (see Figure 2). The term ‘grinding’ is adopted from gaming culture and refers to a laborious and the time-consuming process of earning an in-game achievements. To enter the whitelist, potential buyers have to relentlessly promote, hype up, and invite others to buy into the collection as per requirements set by the collection creators. Since engagement can be easily quantified and measured by friend invites and social shares, social grinding is also competitive.

While some NFT collection creators require user dedication and time, others require a potential buyer to make substantial financial contributions. For example, before it was even launched, one NFT collection, Invisible Friends, required that buyers also had to purchase a physical branded hoodie and beanie for US$150 before they were added to the whitelist (Genc, 2022). Once a whitelist spot is achieved, the buyers can mint a collectible NFT by connecting their crypto wallet to the NFT project website. In some cases, a whitelist also guarantees a cheaper ‘early bird’ price, but this differs from collection to collection. Early access to collectible NFTs rewards the collection adopters with an opportunity to buy into the collection while it is cheaper and then sell when the collection becomes more popular and expensive. But at the same time, it is the social hype and relentless promotion these early adopters generate that make collections valuable. Unlike other forms of assetization, then, the value of an NFT depends more upon the asset purchaser’s active participation in the construction of a future market through promotional practices that are necessarily tied into the experiences of social communities, like personal tastes, hobbies, fandom, or collectibles.

### Trust in me and I will trust in you …

Cryptocurrency enthusiasts often claim that blockchain technologies are ‘trustless technologies’, replacing social trust with more objective, technologically mediated forms of trust. Others note that ‘blockchains don’t actually eliminate trust’, but instead ‘What they do is minimize the amount of trust required from any single actor in the system’ (Bratspies, 2018). Despite the ‘trustless’ label, then, multiple levels of trust are built into blockchain technologies, including trust in developers to build secure software, trust in miners and other actors not to abuse the system’s vulnerabilities, and trust in the decentralized governing actors to spot scams and hacking attempts (Mackenzie & Bērziņa, 2022). NFTs demand a similar layered trust approach: trust in sellers not to sell stolen artwork, trust in artists not to legally pursue copyright protection after selling their artwork as NFTs, and trust in the marketplaces to remove stolen artwork.

Bratspies (2018) argues that the misleading assumptions surrounding the term ‘trustless technology’ make blockchain technologies the perfect vehicles for scams, since trust is placed in potentially questionable social actors within the blockchain governance setup. By design, blockchain technologies were created to bypass government regulations and resist cooption by centralized financial systems. This design includes the anonymity of users involved in transactions, limited supplies of coins or tokens, and the lack of centralized control and oversight. Paradoxically, all principles that make blockchain technologies socially untrustworthy are instead offered as reasons to ‘trust the technology’. Bratspies notes, however, that users rarely interact with the blockchain directly, relying instead on a myriad of third-party tools and entities that are often vulnerable and susceptible to manipulation due to poor maintenance or oversight, as witnessed with the 2022 collapse of the FTX cryptocurrency exchange. As De Filippi et al. (2020) emphasize, ‘decentralized’ is not a synonym for ‘trustless’, since the question of trust does not disappear, but remains in the background. Despite decentralization, for example, blockchain ecosystems, including NFTs, still depend on obscure and often unregulated third-party entities that support blockchain transactions.

In their analysis, Knittel et al. (2019) highlight the continuing importance of social relations as the basis of trust, even when technologically mediated. They look at discussions in the Bitcoin Reddit community in order to understand how the community maintains trust in the cryptocurrency. They find that participation in the community necessitates the adoption of an ideology as a ‘True Bitcoiner’. Such ideology is founded on rejecting the centralized financial system as corrupt and believing that blockchain technologies are truly ‘trustless’ and infallible. Knittel et al. note that the adoption of such an ideology also minimizes the ability to recognize risk and encourages working towards collaborative futures within the community. As Çalışkan (2023) also points out, social or communal legitimation and enforcement does not disappear with the advent of blockchain technologies. Instead, there is a shift in emphasis towards a form of trust in notions—even imaginaries—of decentralization and technological neutrality, which remain some of the most pervasive and utopian driving narratives behind blockchain technology *and* its perceived social and economic value.

The contradiction between the social embeddedness of NFT value and valuation discussed above and the trustless technology of blockchain leads us to suggest that the *social* basis of ‘trustless technology’ is a form of ‘abstracted embeddedness’ (Birch, 2012), entailing the abstracting of intangible social relations (e.g. friendship) as digital things (e.g. metrics, rankings, etc.) (Fourcade & Healy, 2024). In relation to the digital economy, Tkacz (2019) uses the term ‘embedded abstraction’ to differentiate what indebtedness signified in classic economics from the contemporary embedded abstractions like Apple Pay (and other digital finance systems). He notes that this convergence of traditional monetary systems and modern experiential transaction systems is not perfect, but it significantly complicates any critique of digital assets as simple abstractions of social embeddedness. Digital assets constructed via blockchain technologies are still socially embedded, reflecting the argument that an asset can be defined as much by its ‘function’ as its form (Callon, 2021; Fuller, 2013). Abstracted embeddedness necessarily combines both function and form, meaning that it covers both the social experiences and the digital tokens used to represent those experiences. For example, the 2022 Australian Open minted 6,776 ‘Art Ball’ NFTs linking different tiny plots of each tennis court surface to each NFT (Ausopen.com, 2022). As matches were played on the courts, any winning shot was recorded against each plot. The value of the NFT lay in the value placed by individuals on that experience, such as buying a specific NFT linked to a specific part of the court.

Abstracted embeddedness is central to the tokenization of experiences, such as the conversion of ‘interests’, ‘fandom’, or ‘being there’ into an NFT. To be an asset, NFTs depend upon their embeddedness within a social community (Çalışkan, 2023). For example, a community of tennis aficionados who care about tracking tennis statistics, or music fans who are interested in a music group and have a desire to record the experience of attending or participating in that band’s concerts. Tokenization of these social experiences and relations through blockchain technologies turns these social experiences and relations into digital assets, such as NFTs, because they can be made distinct and scarce through the tokenized ownership structure (i.e. fractional). However, while blockchain is technically trustworthy in theory, in practical implementation blockchain technologies can be insecure and vulnerable because of their abstracted embeddedness. For example, as a blockchain grows due to popularity, it takes longer to verify individual blockchain transactions, potentially undermining its technological trustworthiness. In the peak of the Bitcoin frenzy in December 2017, transaction processing times rose to nearly 20 hours per transaction, causing transaction fees to rise significantly. And blockchain scammers can exploit such delays in communication across decentralized databases (Bratspies, 2018).

### Property and entitlement rights

In order to create an NFT, the seller has to initiate the minting process from the chosen marketplace dashboard. For example, Mintable Marketplace sends a request to a MetaMask browser extension, which the user has to accept to proceed. Then, the seller has to prepare an asset listing. This entails choosing a name, subtitle, and description, as well as uploading the artwork and choosing whether they are willing to transfer the copyright to the potential buyer. For all our artworks, we chose not to transfer copyright. Next, we had to choose a transaction style: flat fee or auction. For two of our NFTs, Bridge and Decentraland, we choose auction transaction style and set the expiration date at 7 days. We priced them both with the lowest starting bid of 000.1 ETH (or US$17). For our third NFT, with an image of a pixelated cat, we set the transaction style to flat fee, and priced it at 0.5 ETH or about US$85. If Frye (2022) could sell a stock photo of a bridge for US$100, we thought the cat picture could make at least $85. As we were deciding on the prices for the art pieces, we realized that pricing NFTs is difficult because we are not certain how to estimate the ‘function’ of the images we were auctioning.

As part of our research-creation, we analysed the peculiar characteristics of property and ownership rights and their history in comparison with other assets (see Pistor, 2019). An NFT can be created and uploaded to an NFT marketplace—like the images we produced and uploaded to Mintable Marketplace—but the NFT does not yet exist as an asset *until* a number of bids are collected and the NFT is minted (i.e. added to a blockchain), after determining a winning bid. Here, ownership is embedded in the digital asset itself but only after it is actually sold. Before this, an NFT exists in a state we conceptualize as ‘pre-asset’: An NFT can be created, uploaded, and auctioned, but it does not exist as an asset until it is minted on the blockchain that generates both the digital thing itself and its ownership relations. As such, the digital form and ownership rights constitute a necessary whole with digital assetization (Rotta & Parana, 2022), which contrasts with the assetization of other things (see Birch & Muniesa, 2020, for examples).

In part, NFT creation and ownership are necessarily surrounded by a series of metaphorical abstractions: Users can *mint*, *create*, *upload*, *collect*, *trade*, and *bid on* NFTs, or can *buy* and *own land* in the metaverse. Underneath these metaphors, however, there is no easily definable ‘object’ to own. Even though the digital record is unique on the blockchain, the underlying digital file can be easily reproduced and does not represent exclusive rights of associated functions that could easily be available to others (e.g. images like the ones we created) (Fuller, 2013). Without tokenization via blockchain, the NFT is not ownable or controllable like other assets, which means that its valuation depends upon the transformation of something—especially experiences—into an asset through its very construction as an NFT.

Despite the peculiarities of NFTs as assets, there is considerable optimism about the potential of such blockchain technologies for particular sectors. For example, some scholars argue that NFTs, which enable new, hybrid mechanism for ownership, will help artists assert control over the distribution of their work (Abbate et al., 2022; Trautman, 2021; Whitaker, 2019). Trautman (2021) sees NFTs as a pushback against contemporary exploitative forms of property rights underpinning streaming subscription-based services (e.g. Spotify, Netflix) that legally define customers as licensees, rather than owners, of digital objects. Trautman hopes that NFTs can set a legal precedent as digital ownership mechanisms and believes that future models of ownership of digital and non-digital objects will be shaped by NFTs, meaning that the owner would gain additional privileges or rights with the object (digital or otherwise). Taking this one step further, Whitaker (2019) posits blockchain technologies as not only organizational systems that could help generate value for artists, but also as ways to introduce forms of fractional ownership into artistic entrepreneurship. Here, fractional ownership would enable hybrid ownership of artistic work, which, if managed by a decentralized ledger, could enable a realignment of value across public and private sectors, as well as national jurisdictions (also see Loepfe & Naegeli, 2024).

NFTs should not be conflated or confused with existing intellectual property rights, like copyright. Tokenization does not correspond to copyright (Guadamuz, 2021), in that something can be turned into an NFT without conferring copyright to the NFT owner. Consequently, copyright infringement has been highlighted as an issue for the development of the NFT market. The first precedent of legal action taken against tokenization was in the music industry, when a music producer Damon Dash attempted to tokenize a part of Jay-Z’s album without holding any rights to redistribute the artwork (Guadamuz, 2021). In his defence, Damon Dash explained that he was not trying to sell the artwork, but rather to monetize the ‘interest’ in the record. As Fairfield (2021) notes, NFTs come to represent ‘digital interest’ sold as personal property, reflecting a key element in an emerging experience economy. However, there are no legal procedures in place targeted at determining the applicable jurisdictions and laws suitable for handling NFT disputes, especially when it comes to the ambiguity over whether an NFT represents ‘interest’, or can be considered an adaptation, reproduction, or communication to the public (Guadamuz, 2021).

Besides copyright laws, there are other legal obstacles standing in the way of the treating NFTs like other assets, many of which are related to the socio-technical limitations of blockchain. First, due to the general anonymity of blockchain technologies, it is difficult to legally verify the identities of buyers and sellers, since neither NFT marketplaces nor crypto-wallets record, validate, or trace identifying user information. Second, special terms and conditions of sale (e.g. commercial or merchandising licenses) are not encoded or stored in NFT transactions and have to be legally validated and enforced separately. Third, the digital object sold as an NFT is not stored or recorded on the transaction ledger, so should the server that stores the asset go down, the asset itself becomes inaccessible, even if the NFT token remains valid (see Marlinspike, 2022). Analyzing blockchain’s potential to store licensing information, Guadamuz (2021, p.1375) writes that although, theoretically, it is possible to do, in practice ‘it is difficult to find NFTs that perform any other function’ besides ‘the transfer of the token itself’. When it comes to embedding the digital object into the blockchain (as opposed to providing the link to the digital object, like it is typically done), such transactions are very costly, and therefore not economically viable. NFTs have to undergo full centralization to become legitimate assets, thus abandoning the current implementation of blockchain in favor of a more reliable, centralized database. Such a transition would make it possible to validate, track, and store transactions, verify buyers and sellers, and regulate market speculation.

### Governing NFTs as assets

The governance of NFTs is defined by the specific techno-economic configuration of them as digital assets. After submitting our NFTs to the Mintable Marketplace, for example, we noticed that our seller reputation was instantly set to 5-stars, even though we had not made a single sale or interacted with any potential buyers on the platform. Bodó et al. (2021) argue that in the world of online interactions, the lack of social trust is often mitigated by the establishment of digital trust regimes, like ratings, stars, comments, and other metrics that are supposed to replace the kind of trust that comes from personal experience (see Fourcade & Healy, 2024). Online trust regimes, however, are often based on unclear factors that rarely translate into conventional trustworthiness. By submitting our first NFTs for sale and receiving a 5-star rating, we became, by definition, trustworthy social actors, at least for a while.

The more established NFT community consists of entrepreneurial actors who facilitate digital asset governance through networking, promoting the ‘true NFT enthusiast’ ideology, investing in each other’s ventures, creating demand in the NFT collections, and building up consumer fanbases. NFT projects like Deadfellaz, featuring a series of post-apocalyptic decomposing zombie characters, are more than collections, and function like other branded assets. Deadfellaz own land plots in Decentraland, and bundle their digital asset tokens with 3D avatars usable in the metaverse—and they gift honorary NFTs to celebrities and create fan art and music community contests to further their promotional opportunities.

In the case of many NFT collections, ownership represents membership of an ‘exclusive club’, where, among the immediate benefits of ownership and promises of future integrations with the metaverse, the owners also get to share and shape in-group status and hierarchies. Many NFT collections offer NFT tokens to celebrities who inadvertently (or not) promote exclusive collection memberships by association. To make their assets more lucrative, some NFT collections and communities started offering copyright and IP rights upon sale of the digital assets. It is important to emphasize, however, that this transference of copyright is not legally enforceable. Lawyers have noted that ‘It may come as a surprise to some purchasers that the rights associated with their NFTs do not include the intellectual property rights to the underlying work’ (McMillian, 2021). Furthermore, the same lawyers explicitly state that for purchasing an NFT working with a lawyer is necessary to draft and negotiate intellectual property licencing.

Despite these limitations, the enthusiastic rhetoric about NFTs reconfiguring the legal systems supporting copyright and thereby redefining the systems of ownership resonates with many who place faith in blockchain technologies. However, most NFT enthusiasts are unable to clearly describe the governance dynamics of digital assets like NFTs without resorting to a belief that the ‘market will figure it out’. Frye (2022, p. 139), for example, writes that although there is a logic underlying NFT demand, ‘no one knows what it is yet’. As such, the governance of NFTs and blockchain technology often hinges on narratives of future expectations, whether financial or community-centered, as well as the reconfiguration of techno-economic conditions surrounding the digital asset to ensure that those expectations are achievable (Beckert, 2016).

A particular governance issue with NFTs is how to shift from a Ponzi-like structure to a more conventional marketplace (Callon, 2021). Susman (2022) argues that asking whether NFTs are a Ponzi scheme is the wrong question. Comparing NFT hype to the appeal of expensive brand clothing, he instead argues that the rising popularity of NFT technology is predicated on faith in the lucrative potential of the medium:

The question shouldn’t be whether NFTs are a Ponzi scheme. Of course, they are. Because from designer clothing to houses to stocks, your investment decisions are based on faith. While you may not have faith in NFTs, plenty of people do, and the majority usually wins.

Ultimately, Susman argues that the success of any investment trend is dictated by the faith of enough people. Notably, Ponzi schemes fail not due to the lack of faith of those invested but because of an inability to sustain continuous growth by recruiting new members. Susman’s sentiment, then, echoes the principles of the ‘True Bitcoiner’ ideology observed by Knittel et al. (2019) among the Bitcoin enthusiasts: you have to become a believer to get it. Unlike other more established assets, NFTs are at a liminal stage as an asset class: to govern digital objects as an asset (Birch, 2024) necessitates a continual expansion of the community of people who adhere to the belief that NFTs are both useful *and* lucrative economic assets. A tension, or perhaps self-fulfilling prophecy, follows from this: NFTs are only useful and lucrative economic assets *because* of the growing NFT community who believe that. Where that community collapses or just stutters, then the usefulness and value of NFTs will also collapse or fall.

## Conclusion

Despite renewing our NFT auctions a few times, to date, none of the NFTs we sought to sell were bid on; we could not successfully reproduce Frye’s experiment of selling NFTs without really trying. Through our research-creation process, though, we were able to analyse the decentralized landscape of unregulated blockchain technologies and a parallel digital world characterized by a lack of consumer and creator protections and unregulated by centralized authorities.

A utopian narrative of ‘trustless’ technology underpins the techno-economic dynamics in the digital assetization of NFTs, highlighting how digital assets are different in kind and form than other assets—at least, at present. This digital assetization hinges on the extent to which an asset class can be created out of nothing and made to look like any other investable asset class. Assets are defined by legal entitlements meaning that assets and their value are socially configured, rather than being intrinsic or inherent: This is not different with NFTs. NFTs are different, however, on a number of levels. While they are socially configured like other assets, NFTs are not subject to the same socio-legal arrangements that define their form and function as assets. The current NFT market operates on the assumption of future legal recognition, even though it is simultaneously premised on notions—and even an ideological commitment to—technologically-mediated decentralization. Despite the corporate investment into NFT technologies, then, the hesitancy of lawmakers to define and classify NFTs as an asset class means that digital assetization creates a series of ambiguities.

First, even though there are several potential approaches to applying legal classifications to NFTs, all of them would change the very nature of the digital assets and their distribution. Frye (2022) argues that NFTs can be understood as securities because they act in a similar way to stocks and bonds. However, recognizing NFTs as securities would bring stricter regulations to the existent marketplaces and traders, forcing them to obtain trading licences and creating dependence on mainstream intermediaries (e.g. clearing banks) that blockchain enthusiasts are seeking to avoid. Second, if NFTs are legally redefined as intellectual property, it would hinder the reselling potential of fractional tokens by inserting added transactional complexity, thus subverting the existent marketplace ecosystem. Finally, if NFTs are defined as commodities, then they would be a subject to commodity trading regulations (e.g. *Commodity Exchange Act* in the USA) which would, again, impose stricter regulations on transactions and undermine the principle of market decentralization. Ultimately, being a derivative product of blockchain technologies, NFTs are designed to be resistant to regulation and centralization.

Even though NFTs incorporate ownership history in the tokens themselves, the meaning of such ownership remains interpretive and unclear, since it is currently neither enforceable nor protected. In the NFT market ecology, authenticity becomes an unquestioned abstraction, which helps to drown out problematic issues like copyright infringement, art theft, and other forms of speculative behavior. As such, while NFTs initially excited artistic communities as a game-changing technology that had the potential to rejuvenate creative economies, the latest research shows that digital assetization has not economically favoured artists or creators. As NFT marketplaces gradually became saturated with artistic pieces, market investment shifted towards NFT startups and business ventures that emerged as transactional intermediaries, while early adopters of NFTs recouped their ‘investment’ at the expense of later buyers. In addition to these shifting interests, the techno-economic configuration of NFT markets has not ended up protecting individual creators or ensuring experiential authenticity.

NFTs can be characterized as ‘pre-asset’ since their value is predicated on the act of ownership—when the ownership history is recorded on the ledger once the asset is sold—and the promises of future legal configuration. Unlike other legally configured assets, then, NFTs do not have the essential ‘durability’ necessary to secure future returns (Pistor, 2019), yet they mimic durable assets in other ways. For example, like durable assets, NFTs are bound by temporality and temporal expectations of return: Most NFTs are bought with the intention to ‘flip’ them in the future, when their asset value increases. Like durable assets, NFTs are traded and collected in portfolios and involve input from many organizational actors in order to function: Artists, developers, buyers, sellers, entrepreneurs, journalists as well as enthusiast communities. Ultimately, NFTs are not digital objects free from social relations or governance; they remain, like other assets, configured by an array of techno-economic relations, knowledges, practices, and devices.
